# Experimental Diabetes Alters the Morphology and Nano-Structure of the Achilles Tendon

**DOI:** 10.1371/journal.pone.0169513

**Published:** 2017-01-17

**Authors:** Rodrigo Ribeiro de Oliveira, Rômulo Medina de Mattos, Luciana Magalhães Rebelo, Fernanda Guimarães Meireles Ferreira, Fernanda Tovar-Moll, Luiz Eurico Nasciutti, Gerly Anne de Castro Brito

**Affiliations:** 1 Inter-institutional Doctorate Program in Morphological Science, Federal University of Ceará / Federal University of Rio de Janeiro, Rio de Janeiro, Brazil; 2 Department of Physical Therapy, Federal University of Ceara, Fortaleza, Ceará, Brazil; 3 Tendon Research Group, Fortaleza, Ceará, Brazil; 4 Institute of Biomedical Sciences, Federal University of Rio de Janeiro, Rio de Janeiro, Brazil; 5 Department of Physics, Faculty of Physics, Federal University of Ceara, Fortaleza, Ceará, Brazil; 6 D'Or Institute for Research and Education (IDOR), Rio de Janeiro, Brazil; 7 National Center for Structural Biology and Bioimaging, Federal University of Rio de Janeiro, Rio de Janeiro, Brazil; Medical Clinic, University Hospital Tuebingen, GERMANY

## Abstract

Although of several studies that associate chronic hyperglycemia with tendinopathy, the connection between morphometric changes as witnessed by magnetic resonance (MR) images, nanostructural changes, and inflammatory markers have not yet been fully established. Therefore, the present study has as a hypothesis that the Achilles tendons of rats with diabetes mellitus (DM) exhibit structural changes. The animals were randomly divided into two experimental groups: Control Group (n = 06) injected with a vehicle (sodium citrate buffer solution) and Diabetic Group (n = 06) consisting of rats submitted to intraperitoneal administration of streptozotocin. MR was performed 24 days after the induction of diabetes and images were used for morphometry using ImageJ software. Morphology of the collagen fibers within tendons was examined using Atomic Force microscopy (AFM). An increase in the dimension of the coronal plane area was observed in the diabetic group (8.583 ± 0.646 mm^2^/100g) when compared to the control group (4.823 ± 0.267 mm^2^/100g) resulting in a significant difference (p = 0.003) upon evaluating the Achilles tendons. Similarly, our analysis found an increase in the size of the transverse section area in the diabetic group (1.328 ± 0.103 mm^2^/100g) in comparison to the control group (0.940 ± 0.01 mm^2^/100g) p = 0.021. The tendons of the diabetic group showed great irregularity in fiber bundles, including modified grain direction and jagged junctions and deformities in the form of collagen fibrils bulges. Despite the morphological changes observed in the Achilles tendon of diabetic animals, IL1 and TNF-α did not change. Our results suggest that DM promotes changes to the Achilles tendon with important structural modifications as seen by MR and AFM, excluding major inflammatory changes.

## Introduction

Tendinopathy is a major health problem in people older than 25 years. The main symptom is pain in the tendon that undermines performance. This typically results from excessive use, the severity depending on the magnitude, frequency and duration of the stimulus overloading the tendon [1]. The tendon, in the presence of pathological processes, shows altered morphology. In most cases, it is characterized by intratendinous degeneration and disorganization of collagen fibers. Macroscopically, it presents itself as mucoid degeneration, with friable, disorganized tissue of a brownish color. Microscopically, it is possible to confirm that the structure shows disorganization and micro-ruptures of collagen fibers [2–4]. The tendon tissue loses the parallel organization of its fibers and presents cellular increment. An increase in production of collagen fibers occurs; however, due to their disorganized pattern, the tendon fibers are friable and prone to premature rupture [5,6]. Nevertheless, it is not only the characteristic of stress that may result in tendon injury [7,8].

Two systematic reviews of literature have indicated that there is substantial evidence of a link between Diabetes Mellitus (DM) and tendinopathy [9,10]. Recently, it has been demonstrated that DM leads to modifications in the Achilles tendon which are compatible with chronic tendinopathy. The state of chronic hyperglycemia has been associated with significant increase in mast cells numbers, a vascular hyperplasia in the cross-sectional transverse area of vessels in the Achilles tendons as well as an increase in vascular endothelial growth factor, type 1 collagen, and NF-κB expression when compared to the tendons of control animals [7].

Furthermore, to the manifestations in the structure of tendons in diabetic patients, it has been stated that the biomechanical properties of tendons in diabetic rats have alterations when compared to healthy animals; changes in the visco-elastic capacity of tendons may decrease the limit of energy transmission to the periphery and induce the tendon to premature rupture due to mechanical stress [11,12].

However, regardless of several studies that associate chronic hyperglycemia with tendinopathy, morphological changes observed in MR images, nanostructure, and pro-inflammatory markers have not yet been established. Therefore, the present study has as a hypothesis that the Achilles tendons of the DM group exhibit characteristics of morphological and structural alterations as in classical tendinopathy. This study reveals new information regarding *in vivo* MR images and nanostructure of the Achilles tendon in diabetic rats.

## Materials and Methods

### Animals

Male Wistar rats (*Rattus norvegicus*) were used, with an initial weight between 300 and 350 g, from Federal University of Ceara. The animals were kept in collective, plastic cages (maximum of 5 animals/cage) in an environment with a temperature of 23 ± 1°C, 12-h light/dark cycle and with free access to a maintenance diet (Labina^®^—Purina PetCare Company) and water *ad libitum*. The animals were monitored and assessed daily to ensure that any changes in an animal’s condition were detected early.

The procedures for handling and care of the animals were in accordance with international standards established by the *National Institute of Health Guide for Care and Use of Laboratory Animals* and were approved by the Commission of Ethics in Animal Experimentation—Federal University of Ceara / UFC, under protocol 51/2011.

### Experimental groups and induction to diabetes

The animals were randomly divided in two experimental groups: Control Group—CG (n = 06) consisting of healthy rats; Diabetic Group—DG (n = 06) consisting of rats induced to Diabetes Mellitus.

The experimental diabetes, equivalent to Type I, was induced by intraperitoneal administration of streptozotocin (Sigma Chemical Co., USA) after fasting for 14 h. The streptozotocin (STZ) was diluted in 10 mM sodium citrate buffer at pH 4.5, in a single dose of 60 mg/kg of animal weight, measured carefully in a precision digital scale. Control animals similarly received equivalent dose (60 mg/kg) of sodium citrate buffer solution, and 30 min after treatment, the animals were fed normally [13].

### Blood glucose

Verification of blood glucose occurred at the following stages of the experiment: 1—after the 14 h fast that preceded the induction of diabetes; 2—seven days after induction, aiming to check the inclusion criteria for diabetes, since only animals that had blood glucose levels above 200 mg/dL (Accu-Chek Activ Glucometer kit) were included; 3—on day 24 after diabetes induction, in order to evaluate glycemic expression on the day of tendon collection. Reagent strips were used (Accu-Chek Activ) for determination of blood glucose from a drop of blood from the tip of the animal’s tail.

### In vivo MR images and Morphometry

Magnetic resonance (MR) images were acquired 24 days after induction of DM. The animals were anesthetized with isoflurane (1–2% for maintenance; up to 3% for induction) (E-Z Anesthesia^®^ Systems). The images were acquired in a 7-T MR scanner (7T/210 Horizontal Bore Magnet ASRMRI System, Agilent Technologies). The images of tendons were recorded applying a T1weigthed spin-echo sequence (TR/TE: 350/15 ms; GAP: 0), in the axial plane (FOV: 50 x 80 mm; matrix: 192x192, slice thickness: 1.0 mm; 10 averages), coronal (FOV: 70x85 mm; matrix: 128 x 128, slice thickness: 0.5 mm; 10 averages) and sagittal (FOV: 80 x 50mm; matrix: 128x128, slice thickness: 0.5 mm; 10 averages) before and after the injection of gadolinium.

For each dataset, the images were visually inspected for artifacts. For image processing, MRIcroN software was used, later the Achilles tendon area was measured using ImageJ software. To compare tendon dimensions between the animals of various body size, tendon CSA data were normalized to body weight [7,11]. The assessment of the morphological characteristics and measurement of the area were performed by two experienced researchers and compared between the groups.

For the qualitative analysis of *in vivo* MR images, an additional method of evaluation was performed considering the images before and after the injection of gadolinium, in which the tendon was considered damaged (positive) or normal (negative). To be considered positive, the tendon must show evident disorganization of tissue and/or gadolinium enhancement in the tendinous core.

### Collection of samples of Achilles tendon

Following MR images acquisition, on the twenty-fourth day after the induction of DM, the animals of both groups were anesthetized with xylazine solution (Rompum^®^—Bayer) (10mg/kg) and ketamine hydrochloride (Ketalar^®^) (25 mg/kg), 0.10 ml for each 100 g of weight and an incision was performed in the posterior region of the hind legs to allow collection of the Achilles tendon from its origins and insertions.

### Determination of levels of Interleukin-1 and TNF-α

Quantification of IL1 and TNF-α were conducted by the ELISA method with the Duo Set kits (R&D Systems). Plates of 96 wells were filled with 50 μL of primary antibody diluted in phosphate buffered saline (PBS) and incubated for 18 h at 4°C. The plate was washed three times with 0.05% Tween PBS. Next, 200 μL/well of 1% PBS/BSA were added to block sites, for 1 h at room temperature. After incubation the plates were washed again. The samples and recombinant cytokines in dilution of known concentration were labeled in 100 μL/well, incubated for two hours at 37°C. After washing the plates, 50 μL of biotinylated detection antibody for each cytokine were added for one hour at 37°C. After washing, the plates were incubated with peroxidase-conjugated streptoavidin diluted in PBS 1:200 (50 μL) for 30 min at room temperature. The plates were washed and incubated with a solution of tetramethylbenzidine for 20 min. The reaction occurred with the addition of 25 mL/well of 2N sulfuric acid. The optical density of the samples was determined by an ELISA reader with a 450 nm filter. The cytokine concentrations (pg/mL) found in the tendons were normalized by total protein concentrations.

### Atomic Force Microscopy-AFM

To evaluate the morphology of the collagen fibers and measure the frequency of D interbands of the fibrils tendons were processed and 1 μm-thick transversal histological sections were made and mounted on slides. To capture the images, a steel disk was added and samples were placed in the Atomic Force Multimodal microscope (Digital Instruments, Santa Barbara, CA, USA), equipped with Nanoscope IIIa controller. Samples were measured in both contact and intermittent (tapping) modes of around 0.01 nN attractive force. The data were acquired and the images processed using a scanning system with resonant frequency per probe and using a silicon cantilever (Veeco-Probes) with an integrated, triangular-shaped tip a radius of 15nN. All tendon images were scanned in 512 x 512 size.

The intermittent contact mode (tapping mode) of the AFM was utilized for the assessment of the viscoelastic properties of the fibrils surface. In this mode, the stem oscillated close to its resonance frequency by a small piezoelectric element fixed at the tip of the AFM. The signal acquired from the detectors measured the oscillation motion at the tip of the cantilever, so that generating a phase signal (phase image), and a phase difference (variation in the height of cantilever in the z axis) that was formed from the different interactions between the tip and the sample, indicating the viscoelastic properties of the surface of the tissue. The phase angle (θ) initially considered was 90°, or θ = + 90°, so that, in phase images, the darker shades imply a softer region, and the lighter shades imply a stiffer region.

Spectral analysis (power spectral density) was performed to check the standards of periodicity of D collagen bands in the Y-coordinate. The data were exported to MatLab in order to eliminate backgrounds. Lorentzian Fit was done on the images to obtain the averages of the spacing between the links of the collagen.

### Statistical analysis

To describe the characteristics of the sample descriptive measures were used, such as: measure of central tendency (mean) and dispersion (standard deviation). For comparison of the averages of the numerical variables between the various treatments employed, the Student's t-test was used for independent samples and comparison between the control group and diabetic group. The data were analyzed in the SPSS software. A significance level of 5% was admitted.

## Results

The control animals maintained stable blood glucose levels (below 100 ml/dL) during all the analyses; however, animals in the group induced to DM showed a consistent increase of glucose levels in the measurements seven (352.1 ± 46.3 ml/dL) and twenty-four (423.7 ± 52.9 ml/dL) days after the induction of DM. The group induced to DM represented a significant decrease (p = 0.01) in weight after the induction of DM in the seventh and the twenty-fourth day (221.7 ± 2.5g), when compared with control group (361.2 ± 3.3g).

### Morphometry of in vivo MR images

**Fig 1:**
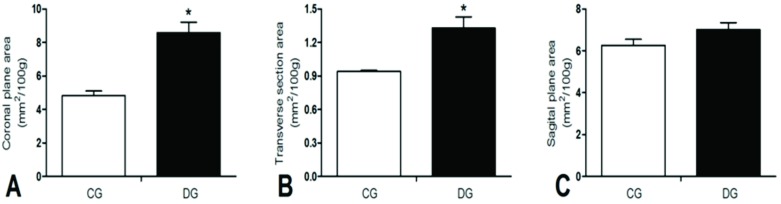
Measurement of Achilles tendon area. A—Area in the coronal plane; B—Area in transverse section; C—Area in the sagittal plane. CG—control group and DG—Diabetic Group. *—p< 0.05.

The diabetic group (8.583 ± 0.646 mm^2^/100g), when compared to the control (4.823 ± 0.267 mm^2^/100g), represented an increase in the size of the coronal plane (p = 0.003) upon evaluating the area of Achilles tendons, normalized by weight. Similarly, the diabetic group (1.328 ± 0.103 mm^2^/100g) in comparison to the control group (0.940 ± 0.01 mm^2^/100g) increased in the transverse section area of the Achilles tendon (p = 0.021). However, no difference was observed between the groups in the normalized area of the tendons in the sagittal plane (Fig 1).

### Qualitative analysis of in vivo MR images before and after gadolinium injection

**Fig 2:**
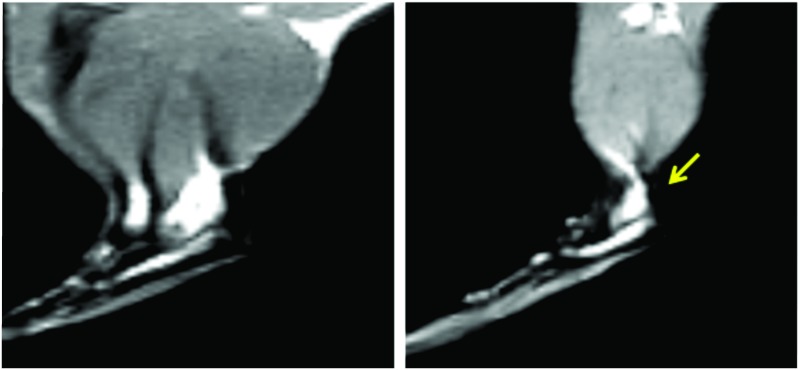
Detail of Sagittal Spin Echo image—T1 weighted of 7T MR images. A—Tendon of the Control Group; B—Tendon of the Diabetic Group showing increased area when compared to the control, in view of the weight. The arrow indicates the location of the Achilles tendon disorganization.

In the qualitative evaluation of the macroscopic organization of the tendons of the control group no changes were noted in the control group (0/6). However, when assessing the DG, 2/6 tendons had alterations in the morphological organization (p = 0.222) as indicated by T1 weighted MR image. No animals, of either group, presented signal enhancement after Gd administration (Fig 2).

### Morphological and topographical characterization with AFM

**Fig 3:**
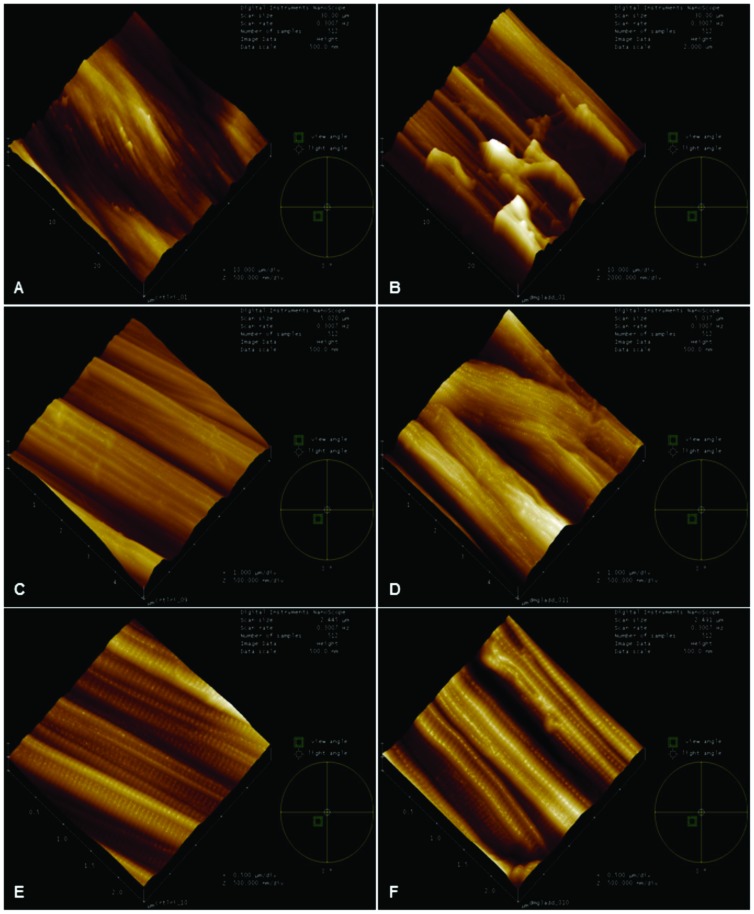
Characterization of surface roughness using Atomic Force Microscopy. 3D representation of the topography of the surface of the fibers and fibrils of the Control Group (A, C and E) and the Diabetic Group (B, D and F) with an area of 30 μm, 5 μm and 2.5 μm. The Control Group (A, C and E) shows nanofibers as uniaxially aligned and well organized fibers. The Diabetic Group (B, D and E), however, shows lack of a uniaxial pattern in collagen fibrillar nano-structure. Fig 3B demonstrates great irregularity of the fiber bundles with breaks in aspect of an abyss with discontinuities. Fig 3D demonstrates the morphological disorganization of the bundles, with modification of the grain direction of the bundles and jagged junctions. Fig 3F shows deformities in the form of bugles collagen fibrils.

The typical structure of the collagen fibers can be observed three-dimensionally by AFM and the topographical evaluations demonstrated well-organized arrangement and a good uniaxial orientation of the healthy Achilles tendons, while, in the Achilles tendons of diabetic animals, there is a notable lack of pattern and disorganization showing changes in fibrillar collagen nano-structure. In the qualitative assessment, it was observed that the collagen fibers had altered their cylindrical shape and exhibited important deformation and discontinuity of the tendon fibers in the diabetic group (Fig 3B, 3D and 3F). Fig 3B demonstrates great irregularity of the fiber bundles with breaks in aspect of an abyss with discontinuities. Fig 3D demonstrates the morphological disorganization of the bundles, with modification of the grain direction of the bundles and jagged junctions. Fig 3F shows deformities in the form of bugles collagen fibrils.

**Fig 4:**
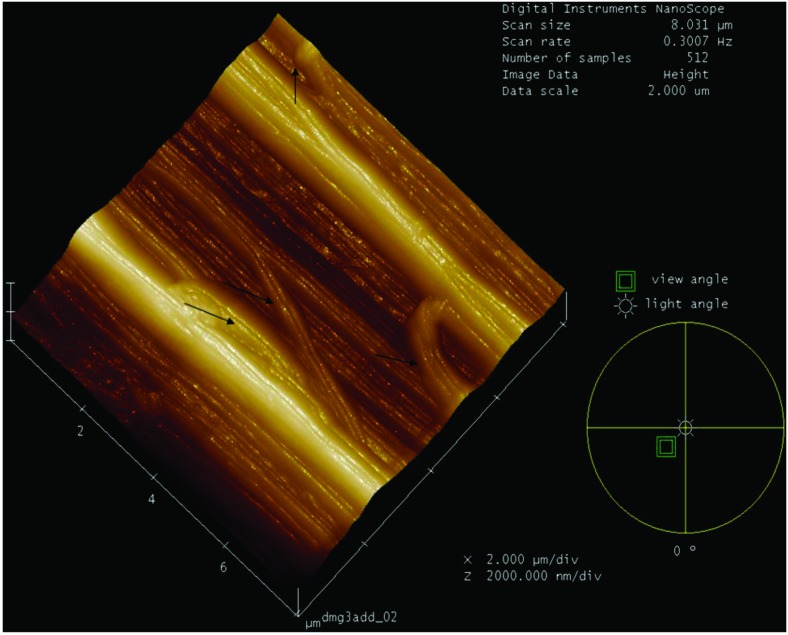
3D representation of the surface topography of the fibrils of the Achilles tendon of a Diabetic Group animal, showing the change in direction of the collagen fibers. The fibers were displaced in opposite directions or exhibited bifurcations, resulting in loss of morphologic and collagen nano-structure features, with changes in the direction of the collagen fibrils indicated by arrows.

The Fig 4 shows the change in direction of the collagen fibers in the Achilles tendon of the diabetic group. The fibers were multidirectional form or bifurcate, resulting in loss of morphological and collagen nanostructure feature.

### Periodicity of Band D

The frequency of D interbands of the fibrils of the diabetic tendon (65.5 ± 0.7) showed no difference from healthy tendons (65.3 ± 2.8), and the frequency of the D band stayed within the benchmark. However, it is important to stress that the fibrils with structural changes were not measured, as they frequently did not present a nano-structure organized in a uniaxial plane or sometimes had rupture of the fibrils and absence of the rings, which did not occur in the healthy group (S1 Fig).

### Phase image of surface topography of fibrils

**Fig 5:**
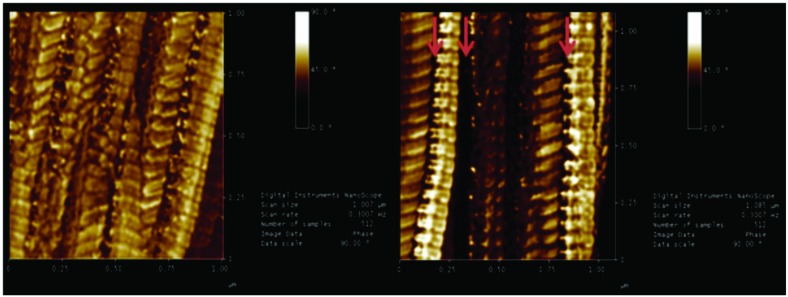
Phase image of surface topography of the fibrils. **A**—Control Group and **B**—Diabetic Group, presenting changes in the interfibril space, where the proteoglycans, GAG chains and the covalent bonds (cross links) are located, the phase angle (dark) was indicated by red arrows. This finding is consistent with more elastic structure. Meanwhile, fibrillar regions showed wide variation of phase angle, when compared to the healthy group, suggesting major changes in the viscoelastic properties of the diabetic tendon.

From the phase images, it was possible to verify that the diabetic group had changes in the mechanical properties, i.e. elasticity measured by AFM, as there was more variation in phase angles in various regions studied. In the interfibril space, where the proteoglycans, glycosaminoglycans (GAG) chains and the covalent bonds (cross links) are located, the phase angle increased. This finding is consistent with more elastic structures. Qualitatively, we could say that the interfibril structures have increased elastic modulus (Young's modulus) in the tendons of diabetic animals. Meanwhile, fibrillar regions showed wide variation of phase angle with some regions more rigid and others more elastic, when compared to the healthy group (Fig 5).

### Inflammatory aspects

**Fig 6:**
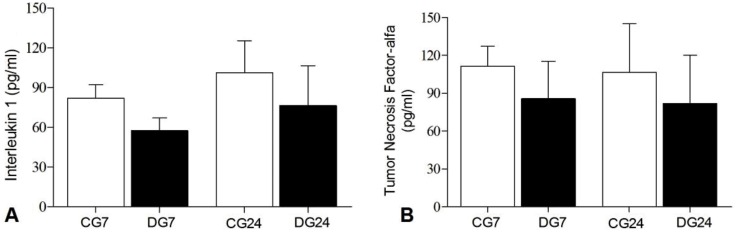
Inflammatory Aspects. Quantification of the concentration of IL-1 **A** and Tumor Necrosis Factor-alfa image **B** through the enzyme-linked immunosorbent assay—ELISA. No statistical differences were found between the groups. Values expressed as mean and standard deviation—P> 0.05.

The Achilles tendons of the diabetic groups did not present increased levels of Interleukin 1—IL1 (Fig 6A) and tumor necrosis factor-alpha—TNF-α (Fig 6B).

## Discussion

This study intends to indicate how the state of chronic hyperglycemia, deriving from experimental Type I Diabetes Mellitus, may influence the homeostatic disproportion of the tendon and, consequently, heads to features of chronic tendinopathy. Therefore, the Achilles tendon was chosen, due to its important biomechanical function, especially in walking, and its superficial position [14]. In addition, some studies have established evidence for biomechanical and histological alterations in the Achilles tendon of diabetic animals [11,15,16] and another study described the morphological alterations in the structure of the Achilles tendon which can predispose the patient to develop a diabetic foot [17].

The induction method of DM was similar to the studies by de Oliveira [7,11,16], a single intraperitoneal administration of STZ solution, considered a verified and well-defined procedure by the literature for the study of complications caused by chronic hyperglycemia similar to Type 1 DM [18].

The increment of the Achilles tendon area is a classic feature of chronic tendinopathy and, typically, is related to overuse of the structure in sports and/or work activities [19,20]. It is often possible to verify the change in tendon thickness in clinical inspection; however, is often revealed by imaging tests, such as ultrasound, computed tomography and MR.

In this study, we observed that in the presence of chronic hyperglycemia, an increment occurred in the Achilles tendon area, detected by *in vivo* MR examination, which was confirmed through analysis of thickness of the tendon by the histomorphometric method. Similar changes of thickness measured by different *in vivo* imaging modalities were previously reported in tendons of various body regions and related to the state of chronic hyperglycemia in human Type 2 diabetics [11,21–24], as well as in animals induced to Type 1 DM using macrometric study [12,17]. However, the MR images and histological feature are more accurate than other assessment techniques for measurements of the tendon area [24]. Therefore, the present study originally used these evaluations for the Achilles tendon to address the theme in experimental Type 1 diabetes as suggested by systematic review [9].

Among the factors that can favor the thickening of the Achilles tendon present in the diabetic group is the disorganized arrangement of collagen fibers and fibrils. This outcome—observed in this investigation with *in vivo* MR and previously via histopathological examinations [7,24]—was confirmed by nanostructural analysis in diabetic animals. In addition to these, the disorganization of collagen was confirmed by another study with atomic force microscopy, where the collagen of rat tails was exposed *in vivo* and *in vitro* to high concentrations of glucose [25], and also by studies evaluating MR images of humans with Type 2 DM [24], reaffirming our considerations.

However, despite observations of the changes in structure and arrangement, the fibrils did not show changes in their axial structure, especially in the periodicity of the D bands. Similarly, other studies with tendinopathy [26] and with analysis of collagen of rat tail tendon exposed to chronic hyperglycemia [25,27] have noted similar findings.

Mechanical modifications presented by the diabetic group in this study were characterized by reduced quality of tendon properties in response to the oscillating stimulus of the tip and cantilever of AFM, notably in the proteoglycans and GAG chains area where the covalent bonds (cross-links) are located. This state could lead to fragile covalent bonds and reduction of stiffness in its matrix, making the tendon friable. The study by Odetti, Aragno et al. (2000) [25] asserted that these changes can be explained by the presence of non-enzymatic glycation (Advanced Glycated End-Products: AGE) in the collagen fibrils in diabetics. In turn, the increasing complacency of linking structures between the collagen fibrils, observed in our study, can explain the mechanical changes and early failure found in diabetic animals by investigations with mechanical traction tests of the Achilles tendon [11,16] and patellar tendon [12].

To establish an inflammatory response, tenocytes produce pro-inflammatory cytokines, such as IL1 and TNF-α, which in turn stimulate the synthesize collagen [28]. Pro-inflammatory cytokines stimulate the expression of metalloproteinases (MMP1, MMP3, MMP 13) which degrade the extracellular matrix (ECM) of tendons. Despite the changes in the ECM of the Achilles tendon of diabetic animals observed in this study, there was no increase in IL1 and TNF-α levels in animals seven and 24 days after induction of DM. In fact, the inflammatory state has not been found in conditions of chronic tendinopathy [29,30]. On the other hand, it is important to remember that the absence of inflammatory mediators in the phases investigated does not mean that they were not present in early stages. However, chronic tendinopathy can occur with little or no acute inflammatory expression [31].

This study evaluates the relationship of DM with changes to the Achilles tendon overall and does not establish causality of the phenomena presented. *In vitro* and *in vivo* analyses at different points in time after induction can help clarify the cascades that modify the tendon of the subject with DM. There is a need, however, to conduct studies that could better clarify the causal pathways of the changes to the Achilles tendon presented by diabetics.

## Conclusion

Chemically induced Diabetes Mellitus promotes changes to the Achilles tendon with structural modifications that are compatible with the process of chronic tendinopathy. This is evidenced by the fact that in the presence of a state of chronic hyperglycemia, such changes occurred as thickening of the tendon with disordered arrangement of collagen fibers and fibrils as well as alteration of mechanical properties on the nanoscale when compared to tendons of healthy animals. However, despite the changes observed in the ECM of the Achilles tendon of diabetic animals, increases in IL1 and TNF-α were not observed in animals at seven and 24 days after induction of DM. It is believed that these changes in structural properties may predispose the Achilles tendon to injury and, consequently, to premature rupture.

## Supporting Information

S1 Fig The frequency of D interbands of the fibrils of the diabetic tendon. It is important to stress that the fibrils with structural changes were not measured, as they frequently did not present a nano-structure organized in a uniaxial plane or sometimes had rupture of the fibrils and absence of the rings.
(DOCX)

## Abbreviations

AFM: Atomic Force Microscope
AGE: Advanced Glycated End-Products
CG: Control Group
DG: Diabetic Group
DM: Diabetes mellitus
ECM: Extracellular matrix
GAG: Glycosaminoglycans
IL1: Interleukin 1
MMP: Metalloproteinases
MR: Magnetic resonance
PBS: phosphate buffered saline
STZ: Streptozotocin
TNF-α: Tumor necrosis factor-alpha

## References

[1] Bohm S, Mersmann F, Arampatzis A (2015) Human tendon adaptation in response to mechanical loading: a systematic review and meta-analysis of exercise intervention studies on healthy adults. Sports Med Open 1: 7. 27747846 10.1186/s40798-015-0009-9 PMC4532714

[2] Zhang ZJ, Ng GY-f, Lee WC, Fu SN (2014) Changes in morphological and elastic properties of patellar tendon in athletes with unilateral patellar tendinopathy and their relationships with pain and functional disability. PloS one 9: e108337 10.1371/journal.pone.0108337 25303466 PMC4193737

[3] Glazebrook MA, Wright JR Jr., Langman M, Stanish WD, Lee JM (2008) Histological analysis of achilles tendons in an overuse rat model. J Orthop Res 26: 840–846. 10.1002/jor.20546 18183626

[4] Nakama LH, King KB, Abrahamsson S, Rempel DM (2005) Evidence of tendon microtears due to cyclical loading in an in vivo tendinopathy model. J Orthop Res 23: 1199–1205. 10.1016/j.orthres.2005.03.006 16140201

[5] Sharma P, Maffulli N (2005) Tendon injury and tendinopathy: healing and repair. The Journal of Bone & Joint Surgery 87: 187–202. 15634833 10.2106/JBJS.D.01850

[6] Maffulli N, Kader D (2002) Tendinopathy of tendo achillis. J Bone Joint Surg Br 84: 1–8. 10.1302/0301-620x.84b1.12792 11837811

[7] de Oliveira RR, Martins CS, Rocha YR, Braga AB, Mattos RM, et al (2013) Experimental diabetes induces structural, inflammatory and vascular changes of Achilles tendons. PloS one 8: e74942 10.1371/journal.pone.0074942 24130676 PMC3794027

[8] Riley G (2004) The pathogenesis of tendinopathy. A molecular perspective. Rheumatology 43: 131 10.1093/rheumatology/keg448 12867575

[9] de Oliveira R, Lemos A, de Castro Silveira P, da Silva R, de Moraes S (2011) Alterations of tendons in patients with diabetes mellitus: a systematic review. Diabetic Medicine 28: 886–895. 10.1111/j.1464-5491.2010.03197.x 21749441

[10] Ranger TA, Wong AM, Cook JL, Gaida JE (2015) Is there an association between tendinopathy and diabetes mellitus? A systematic review with meta-analysis. British journal of sports medicine: bjsports-2015-094735. 10.1136/bjsports-2015-094735 26598716

[11] de Oliveira RR, de Lira KD, de Castro Silveira PV, Coutinho MP, Medeiros MN, et al (2011) Mechanical Properties of Achilles Tendon in Rats Induced to Experimental Diabetes. Ann Biomed Eng 39: 1528–1534. 10.1007/s10439-011-0247-z 21225344

[12] Fox AJS, Bedi A, Deng XH, Ying L, Harris PE, et al (2011) Diabetes mellitus alters the mechanical properties of the native tendon in an experimental rat model. Journal of Orthopaedic Research. 10.1002/jor.21327 PMC5243138 21246619

[13] Dall'Ago P, Silva VOK, De Angelis KLD, Irigoyen MC, Fazan R Jr., et al (2002) Reflex control of arterial pressure and heart rate in short-term streptozotocin diabetic rats. Brazilian Journal of Medical and Biological Research 35: 843–849. 12131926 10.1590/s0100-879x2002000700013

[14] Lichtwark GA, Barclay CJ (2010) The influence of tendon compliance on muscle power output and efficiency during cyclic contractions. The Journal of experimental biology 213: 707–714. 10.1242/jeb.038026 20154185

[15] Batista F, Nery C, Pinzur M, Monteiro AC, de Souza EF, et al (2008) Achilles tendinopathy in diabetes mellitus. Foot Ankle Int 29: 498–501. 18510903 10.3113/FAI-2008-0498

[16] de Oliveira RR, Bezerra MA, de Lira KDS, Novaes KA, Teixeira MFHBI, et al (2012) Aerobic physical training restores biomechanical properties of Achilles tendon in rats chemically induced to diabetes mellitus. Journal of Diabetes and its Complications 26: 163–168. 10.1016/j.jdiacomp.2012.03.017 22520401

[17] Giacomozzi C, D'Ambrogi E, Uccioli L, Macellari V (2005) Does the thickening of Achilles tendon and plantar fascia contribute to the alteration of diabetic foot loading? Clin Biomech (Bristol, Avon) 20: 532–539. 10.1016/j.clinbiomech.2005.01.011 15836941

[18] Szkudelski T (2001) The mechanism of alloxan and streptozotocin action in B cells of the rat pancreas. Physiol Res 50: 537–546. 11829314

[19] Karjalainen PT, Soila K, Aronen HJ, Pihlajamäki HK, Tynninen O, et al (2000) MR imaging of overuse injuries of the Achilles tendon. American Journal of Roentgenology 175: 251–260. 10.2214/ajr.175.1.1750251 10882283

[20] Paavola M, Kannus P, Jarvinen TA, Khan K, Jozsa L, et al (2002) Achilles tendinopathy. J Bone Joint Surg Am 84-A: 2062–2076. 12429771 10.2106/00004623-200211000-00024

[21] Akturk M, Karaahmetoglu S, Kacar M, Muftuoglu O (2002) Thickness of the supraspinatus and biceps tendons in diabetic patients. Diabetes Care 25: 408. 10.2337/diacare.25.2.408 11815529

[22] Akturk M, Ozdemir A, Maral I, Yetkin I, Arslan M (2007) Evaluation of Achilles tendon thickening in type 2 diabetes mellitus. Exp Clin Endocrinol Diabetes 115: 92–96. 10.1055/s-2007-955097 17318767

[23] Bolton NR, Smith KE, Pilgram TK, Mueller MJ, Bae KT (2005) Computed tomography to visualize and quantify the plantar aponeurosis and flexor hallucis longus tendon in the diabetic foot. Clin Biomech (Bristol, Avon) 20: 540–546. 10.1016/j.clinbiomech.2004.12.007 15836942

[24] Papanas N, Courcoutsakis N, Papatheodorou K, Daskalogiannakis G, Maltezos E, et al (2009) Achilles tendon volume in type 2 diabetic patients with or without peripheral neuropathy: MRI study. Exp Clin Endocrinol Diabetes 117: 645–648. 10.1055/s-0029-1224121 19834869

[25] Odetti P, Aragno I, Rolandi R, Garibaldi S, Valentini S, et al (2000) Scanning force microscopy reveals structural alterations in diabetic rat collagen fibrils: role of protein glycation. Diabetes/metabolism research and reviews 16: 74–81. 10751746 10.1002/(sici)1520-7560(200003/04)16:2<74::aid-dmrr80>3.0.co;2-1

[26] Yoo SD, Choi S, Lee G-J, Chon J, Jeong YS, et al (2012) Effects of extracorporeal shockwave therapy on nanostructural and biomechanical responses in the collagenase-induced Achilles tendinitis animal model. Lasers in medical science 27: 1195–1204. 10.1007/s10103-011-1049-0 22274874

[27] Gonzalez AD, Gallant MA, Burr DB, Wallace JM (2014) Multiscale analysis of morphology and mechanics in tail tendon from the ZDSD rat model of type 2 diabetes. Journal of biomechanics 47: 681–686. 10.1016/j.jbiomech.2013.11.045 24360194 PMC3918737

[28] Le J, Weinstein D, Gubler U, Vilcek J (1987) Induction of membrane-associated interleukin 1 by tumor necrosis factor in human fibroblasts. The Journal of Immunology 138: 2137–2142. 3494060

[29] Alfredson H, Thorsen K, Lorentzon R (1999) In situ microdialysis in tendon tissue: high levels of glutamate, but not prostaglandin E2 in chronic Achilles tendon pain. Knee surgery, sports traumatology, arthroscopy 7: 378–381. 10.1007/s001670050184 10639657

[30] Kannus P, Natri A (1997) Etiology and pathophysiology of tendon ruptures in sports. Scand J Med Sci Sports 7: 107–112. 9211611 10.1111/j.1600-0838.1997.tb00126.x

[31] Maffulli N, Renström P, Leadbetter WB (2005) Tendon Injuries: Springer.

