# Supplementary material for: Experimental Diabetes Alters the Morphology and Nano-Structure of the Achilles Tendon
Source: PLoS One. 2017 Jan 17;12(1):e0169513. doi: 10.1371/journal.pone.0169513 (PMC5240962; doi:10.1371/journal.pone.0169513)
Supplement: S1 Fig — It is important to stress that the fibrils with structural changes were not measured, as they frequently did not present a nano-structure organized in a uniaxial plane or sometimes had rupture of the fibrils and absence of the rings. (DOCX) [file pone.0169513.s001.docx]

*
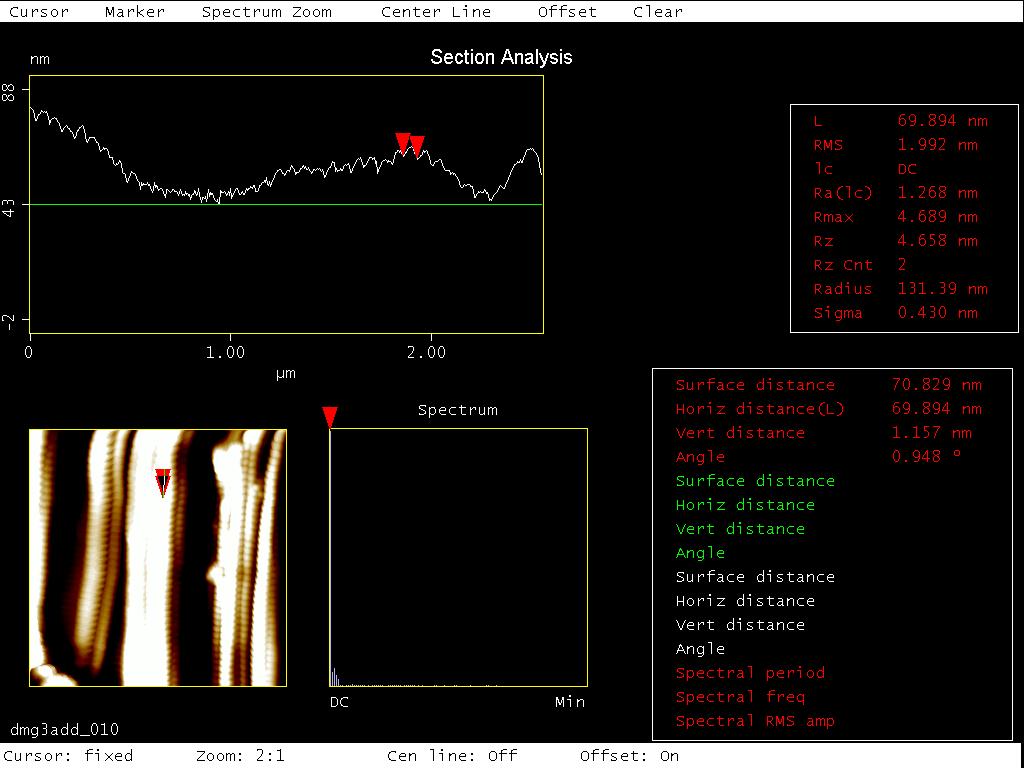
*

***Supplementary Figure 01*** *- The frequency of D interbands of the fibrils of the diabetic tendon.* *It is important to stress that the fibrils with structural changes were not measured, as they frequently did not present a nano-structure organized in a uniaxial plane or sometimes had rupture of the fibrils and absence of the rings.*
